# Influence of Altered Microbes on Soil Organic Carbon Availability in Karst Agricultural Soils Contaminated by Pb-Zn Tailings

**DOI:** 10.3389/fmicb.2018.02062

**Published:** 2018-08-31

**Authors:** Qiang Li, Chang Liu, Xiaohong Wang, Zhenjiang Jin, Ang Song, Yueming Liang, Jianhua Cao, Werner E. G. Müller

**Affiliations:** ^1^Key Laboratory of Karst Dynamics, Ministry of Land and Resources & Guangxi Zhuang Autonomous Region, Institute of Karst Geology, Chinese Academy of Geological Sciences, Guilin, China; ^2^The International Research Center on Karst under the Auspices of UNESCO, Guilin, China; ^3^ERC Advanced Investigator Grant Research Group at Institute for Physiological Chemistry, University Medical Center of the Johannes Gutenberg University Mainz, Mainz, Germany; ^4^Environmental Sciences and Engineering College, Guilin University of Technology, Guilin, China

**Keywords:** soil organic carbon, karst surface soil, microbial community, long-term acclimation, Pb-Zn tailings

## Abstract

Soil organic carbon (SOC) availability is determined via a complex bio-mediated process, and Pb-Zn tailings are toxic to the soil microbes that are involved in this process. Here, Pb-Zn-tailings- contaminated karst soils with different levels (paddy field > corn field > citrus field > control group) were collected to explore the intrinsic relationship between Pb-Zn tailings and microbes due to the limited microbial abundance in these soils. The SOC concentration in the paddy fields is the highest. However, based on the soil microbial diversity and sole-carbon-source utilization profiles, the rate of SOC availability, McIntosh index, Shannon-Wiener diversity index, Simpson’s diversity index and species richness are the lowest in the rice paddy soils. According to the results of Illumina sequencing of the 16S rRNA gene, Acidobacteria and Proteobacteria are the dominant phyla in all samples, accounting for more than 70% of the reads, while the majority of the remaining reads belong to the phyla Verrucomicrobia, Chloroflexi, Actinobacteria, Bacteroidetes, and Nitrospirae. We also observed that their class, order, family, genus and operational taxonomic units (OTUs) were dependent on SOC availability. Pearson correlation analysis reveals that L-asparagine utilization profiles show significant positive correlation with OTUs 24, 75, and 109 (*r* = 0.383, 0.350, and 0.292, respectively), and malic acid utilization profiles show significant positive correlation with OTUs 4, 5, 19, 27 (Bradyrhizobium), 32 (Burkholderia), 75 and 109 (*r* = 0.286, 0.361, 0.387, 0.384, 0.363, 0.285, and 0.301, respectively), as also evidenced by the redundancy analysis (RDA) biplot and heat map. These results indicate that the most abundant groups of bacteria, especially the uncultured facultative Deltaproteobacteria GR-WP33-30 (OTU 24), after long-term acclimation in heavy metal-contaminated soil, are associated with the variance of labile carbon source such as L-asparagine and may have considerable control over the stability of the vast SOC pool in karst surface soils with different agricultural land-use practices. These findings can expand our understanding of global soil-carbon sequestration and storage via changes in microbial community structure of the most abundant species.

## Introduction

Almost 3000 Pg C is stored as soil organic carbon (SOC) (Köchy et al., 2015), which is equivalent to three times the carbon content of the Earth’s atmosphere (Pries et al., 2017). Mineralization and availability of C stimulate climate change; mineralization is a complex bio-mediated reaction in which organic substrates are converted to living biomass and mineral residues (Zhang et al., 2008). Consequently, soil microorganisms, which play an important role in the soil carbon cycle as suitable predictors of transformations involved in the C cycling in soils, account for 80% of the SOC mineralization and variance in availability (Trivedi et al., 2013; Tardy et al., 2015). Previous studies have shown that soil microbial communities and diversity are affected by pH (Rousk et al., 2009), land use (Tardy et al., 2015), temperature (Pries et al., 2017), carbon supply (Fontaine et al., 2007; Whitman et al., 2016), and heavy metals (Clemente et al., 2006; Li et al., 2015; Zhang et al., 2016).

In addition, the SOC pool can be divided into two parts: a labile and a stabilized carbon pool. Labile organic carbon components [microbial biomass carbon (MBC), dissolved organic carbon (DOC), and particulate organic carbon (POC)] are especially important because they are more vulnerable to climatic change (Luo et al., 2017) and are associated with bioavailability of SOC (Bernal et al., 2016; Rousk et al., 2016). Moreover, labile carbon is highly active and sensitive to microorganisms and highly susceptible to oxidation and decomposition (Yang et al., 2017). However, according to the regulatory gate hypothesis, which was proposed based on simulation experiments in the lab (Kemmitt et al., 2008), the rate-limiting step in the mineralization of humified soil organic matter is governed by a biological processes; SOC mineralization is independent of the size, community structure and specific activity of soil microorganisms (Kemmitt et al., 2008; Brookes et al., 2017). Based on the above mentioned studies, it is unclear whether soil microbial communities change with SOC availability when the microbial environment changes, especially in mine-tailings-contaminated soil.

It is known that the mine tailings, which are produced during the extraction and processing of metalliferous ores, present serious environmental concerns because they usually contain high concentrations of heavy metals. Previous studies have shown that post-mining soils have generally low microbial diversity and biomass (de Quadros et al., 2016) due to the toxicity of heavy metals to microorganisms (Clemente et al., 2006; Li et al., 2015; Zhang et al., 2016). Therefore, mine-tailings-contaminated soil can be an ideal medium to study and discuss the intrinsic relationship between soil microorganisms and SOC. Moreover, several studies (Yarwood et al., 2015; Hui et al., 2017) have examined the influence of vegetation on soil microbial communities, though little is known about which is the determining influence of mine-tailings-contamination or vegetation on soil microbial composition. Our previous research revealed that the abundance of cultured soil microorganisms in karst soils with severe Pb-Zn-tailings contamination was lower than that in karst soils with less Pb-Zn-tailings contamination; according to the results of a Biolog Ecoplate assay, the SOC mineralization rate was retarded in karst soils with severe Pb-Zn-tailings contamination (Li et al., 2015). Unfortunately, our previous study did not include appropriate control groups with weak Pb-Zn-tailings contamination (or no contamination) (Li et al., 2015). Moreover, more than 99% of the microorganisms from familiar but poorly characterized soil environments resist laboratory cultivation (Kaeberlein et al., 2002). The inclusion of control groups and uncultured microorganisms in the study should help us better understand the phenomenon mentioned above.

Bacteria and fungi are often the main constituents of soil microbial communities, and fungi are less resistant than bacteria to heavy metal contamination (Jin et al., 2015; Mossa et al., 2017). It is necessary to test the influence of Pb-Zn-tailings-contamination on microbial communities and the microbial feedback to SOC availability. In this context, we hypothesized that (i) toxic Pb-Zn tailings alter the structure and/or function of soil microbial communities; (ii) the altered structure and/or function of soil microbial communities in turn affect SOC availability and alter the stability of the SOC in surface soils of Pb-Zn-tailings-contaminated karst regions; and (iii) the altered structure and/or function of soil microbial communities can be used as a key to understand the process described above. To reduce this knowledge gap, we mainly investigated the effect of toxic Pb-Zn tailings on variance in SOC availability and soil microbial activity under long-term field conditions by using real-time PCR (Denman and McSweeney, 2006) and high-throughput community sequencing data (Li et al., 2017); the V3–V5 region (515F-909R for bacteria and archaea) of the 16S rRNA genes (Tamaki et al., 2011) was amplified and was used for taxonomic analyses of their communities in our soil samples. Based on the results of this study, the large 16S rRNA gene dataset coupled with the ecological features of these soil samples will help us understand how these communities change with SOC availability in Pb-Zn-tailings-contaminated surface soils of karst regions.

## Materials and Methods

### Experimental Design and Soil Sampling

The soil samples were collected from Sidi Village, which is situated in a typical karst depression with approximately 0.5 km^2^ of arable land. As we have previously reported, this land was contaminated by the leakage of deposited Pb-Zn tailings in the 1970s due to the collapse of a poorly constructed dam for the collection of Pb-Zn tailings during storm floods; subsequently, the contaminated farmland was directly plowed for farming activities (Jin et al., 2015; Li et al., 2015). After almost 40 years of agricultural utilization, soil conditions in this region present stability. We selected typical contaminated land that is currently in use; the land can be divided into three types of fields: evergreen field with C3 citrus plants, therophyte field with C3 rice plants and therophyte field with C4 corn plants. In addition, due to the limited land with karst characteristics in this region, the other un-plowed fields with C3 plants, which were not directly contaminated with Pb-Zn tailings, were selected as the control group (CK).

**FIGURE 1:**
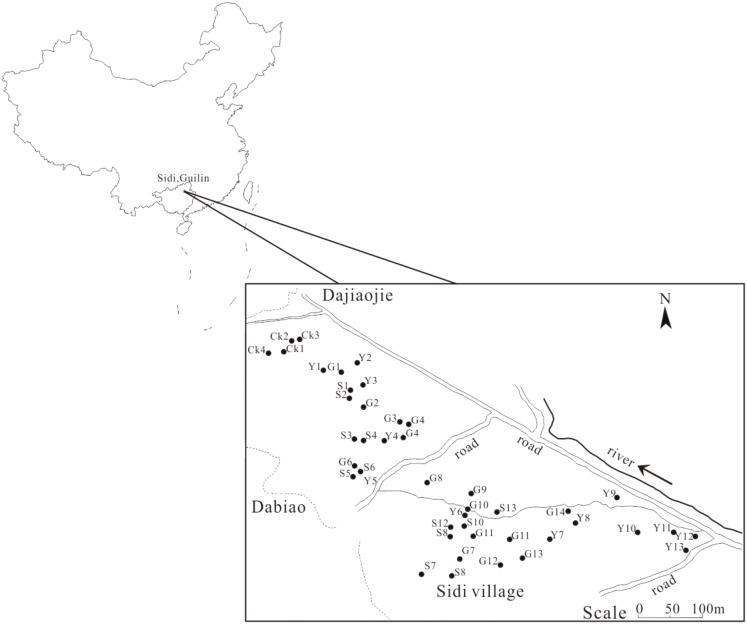
The sampling location in Sidi Village. Y, corn field; S, paddy field; G, citrus field; CK, control group.

According to our experimental design, 13 soil samples from paddy fields, 13 soil samples from corn fields, 14 soil samples from citrus fields and 4 CK soil samples were collected in June 2015. The sampling sites are marked in **Figure 1**. Each soil sample (500 g) from three soil cores that were 5 cm in diameter and 20 cm in depth was thoroughly mixed. The soil samples were sieved through a 0.2 mm mesh to remove all rocks and roots. The homogenized samples were stored at 4°C for subsequent analysis.

### Determination of Soil Parameters

Three replicate sieved soil samples were used to determine the physicochemical properties of the soil. The SOC, DOC, MBC, pH, total nitrogen (TN), available nitrogen (AN), and cation exchange capacity (CEC) were analyzed as described by Jin et al. (2015). POC content was determined by dry combustion in a LECO furnace (Leco Corp., United States) (Merry and Spouncer, 1988). Total and available [diethylene triamine pentaacetic acid (DTPA)-extractable] Pb, Cd, Zn, and Cu (Feng et al., 2005; Li et al., 2015) levels were measured at the Institute of Karst Geology, CAGS, by using flame atomic absorption spectrophotometry (Zeenit 700P, Germany). Biolog EcoPlates (Biolog Inc., United States) were used to evaluate the metabolic activities of microbial communities and the capacities of these communities to utilize different carbon sources; consequently, the McIntosh index, Shannon-Wiener diversity index, Simpson’s diversity index, Shannon’s evenness index and species richness were calculated (Li et al., 2015). The average values are summarized in **Table 1**, and further details about the soil parameters can be found in **Supplementary Table S1**.

**Table 1:** Soil characteristics of Pb-Zn-tailings-contaminated karst soil in Sidi Village.

Land-use type	CK	Corn field	Citrus field	Paddy field
Total-Pb	28.02 ± 8.38B^∗^	1262.5 ± 188.80A	719.41 ± 185.64 A	1388.42 ± 213.24 A
Total-Zn	137.85 ± 16.31 B	1523.22 ± 129.69 A	1291.11 ± 217.19 A	1789.40 ± 190.05 A
Total-Cu	24.58 ± 9.69 B	196.93 ± 28.79 AB	215.73 ± 50.54 A	245.81 ± 27.80 A
Total-Cd	29.37 ± 17.78 B	59.12 ± 5.10 AB	50.77 ± 12.06 AB	81.37 ± 10.34 A
DTPA-Pb	7.98 ± 0.69 B	527.62 ± 66.89 A	355.98 ± 24.46 A	477.36 ± 44.11 A
DTPA-Zn	9.38 ± 0.23 B	326.81 ± 22.38 AB	280.09 ± 49.65 AB	569.14 ± 22.59 A
DTPA-Cu	0.31 ± 0.03 B	30.55 ± 3.53 A	21.14 ± 2.47 A	35.48 ± 3.90 A
DTPA-Cd	4.48 ± 0.13 C	4.64 ± 0.04 BC	4.83 ± 0.06 AB	4.99 ± 0.04 A
TN	0.60 ± 0.09 A	1.17 ± 1.79 A	0.60 ± 0.14 A	0.78 ± 0.24 A
AN	92.47 ± 7.00 B	100.56 ± 6.44 AB	92.81 ± 5.59 B	121.87 ± 8.41 A
CEC	8.50 ± 0.64 A	8.20 ± 1.33 A	7.81 ± 1.54 A	10.03 ± 2.60 A
pH	5.20 ± 0.88 B	5.60 ± 0.51 AB	5.18 ± 0.49 B	5.91 ± 0.38 A
SOC	23.01 ± 3.34 AB	19.37 ± 2.17 BC	14.08 ± 2.99 C	26.84 ± 2.40 A
DOC	165.35 ± 29.39 B	209.97 ± 19.72 B	145.78 ± 8.200 B	223.19 ± 33.41 A
POC	4.13 ± 1.18 B	4.76 ± 0.38 AB	4.73 ± 0.50 AB	7.35 ± 0.82 A
MBC	477.42 ± 41.34 A	412.31 ± 11.00 AB	268.37 ± 13.40 B	249.36 ± 6.54 B
Bacteria	9.28 × 10^10^ ± 1.36 × 10^10^ AB	1.13 × 10^11^ ± 4.64 × 10^10^ A	1.93 × 10^10^ ± 1.06 × 10^10^ B	1.64 × 10^11^ ± 1.12 × 10^10^ A
Fungi	8.95 × 10^7^ ± 8.81 × 10^6^ A	4.34 × 10^7^ ± 1.23 × 10^6^ AB	5.05 × 10^7^ ± 6.29 × 10^6^ AB	2.44 × 10^7^ ± 1.25 × 10^6^ B

*^∗^Data are means ± standard deviations (13 paddy soil samples, 13 corn field soil samples, 14 citrus field soil samples, and 4 CK soil samples). Statistical pairwise multiple comparisons of homogenous data were carried out by the Tukey test: means with the same letter in the same line are not significantly different at P < 0.05. The unit for AN; DOC; POC; MBC; total Pb, Zn, Cu, and Cd; and available Pb, Zn, Cu, and Cd is mg kg^−1^. The unit for TN and SOC is g kg^−1^. The unit for CEC is cmol kg^−1^. The unit for bacteria and fungi is copies g^−1^ of dry soil. DTPA, DTPA-extractable.*

### DNA Extraction and Quantitative PCR

DNA extraction was performed using a Power Soil DNA Isolation Kit (Mo Bio Laboratories Inc., United States) according to the manufacturer’s instructions.

Real-time PCR assays of the 16S rRNA gene, as a marker of bacteria, and the 18S rRNA gene, as a marker of fungi, were carried out in a volume of 25 μL. The assay mixture contained μL of Green-2-Go qPCR MasterMix (Sangon Biotech Co., Ltd, China), 1 μL of primer (10 μmol μL^−1^), 9.5 μL of distilled water, and 1 μL of template DNA (at 5 ng μL^−1^). Thermal cycling conditions for 16S rRNA gene amplification were as follows: an initial step of 95°C for 3 min; 39 cycles of 95°C for 60 s, 56°C for 60 s, and 72°C for 60 s; and 72°C for 5 min. Thermal cycling conditions for 18S rRNA gene amplification were as follows: an initial step of 95°C for 15 min; 39 cycles of 95°C for 60 s, 56°C for 60 s, and 72°C for 60 s; and 72°C for 5 min. The primers for 16S rRNA gene amplification were 338F and 518R, as reported by Li et al. (2017). The primers for 18S rRNA gene amplification were a fungus-specific primer (5′-ATTCCCCGTTACCCGTTG-3′) and NSI (5′-GTAGTCATAT GCTTGTCTC-3′), which were designed by May et al. (2001). Thermal cycling, fluorescence data collection and data analysis were carried out with a CFX96 Touch Real-Time PCR Detection System (Bio-Rad, United States) according to the manufacturer’s instructions. The relationship between *C*_t_ and the log of the starting concentration was linear (*R*^2^ > 0.99). The amplification efficiency was 90 ∼ 105%.

### 16S rRNA Gene Sequencing

For HiSeq sequencing, PCR primers 515F (5′-CTACCGATTGCGGTGYCAGCMGCCGCGGTA-3′) and 909R (5′-CCCCGYCAATTCMTTTRAGT-3′) were used to amplify the V3–V5 region of the 16S rRNA genes (Tamaki et al., 2011). The PCR products of the V3–V5 region of the 16S rRNA genes were purified by using the TIANquick Maxi Purification Kit [TIANGEN Biotech (Beijing) Co., Ltd., China]. Then, 16S rRNA gene sequencing was performed with the 2 × 250-bp paired-end strategy on the Illumina HiSeq 2500 platform (Illumina Inc., United States) at the Chengdu Institute of Biology, Chinese Academy of Sciences.

The raw sequence reads were deposited in the NCBI Sequence Read Archive under the accession number PRJNA418926.

### Statistical Analyses

All *P*-values for the relative abundances of the taxa identified from the 16S rRNA gene were calculated using paired two-tailed *t*-tests, which were processed according to the method described by Benjamini et al. (2006). The 16S rRNA gene sequencing data were visualized using principal coordinates analysis (PCoA) plots generated with the EMPeror software package (Vázquez-Baeza et al., 2013). Partial Least Squares Path Modeling (PLS-PM) was used to explore the relationships among microbial communities and soil factors by using R package^1^ (Sanchez, 2013).

The quantitative PCR results are summarized in **Supplementary Table S1** (Supporting Information). For each soil sample, the average values and standard errors were calculated from seven replicate quantitative PCR results.

Moreover, multiple pairwise comparisons of soil parameters were performed using the Tukey test with a significance level of 0.05. The normalized values were calculated as described by Jin et al. (2013). Correlation analyses were performed using the Pearson correlation method (one-tailed *t*-test) with significance defined as *P* < 0.05 by using SPSS 13.0 software for Windows XP (IBM, United States). For principal component analysis (PCA) and redundancy analysis (RDA), CANOCO for Windows 4.5 was used.

## Results and Discussion

### Dateset Sequencing

Low quality sequences (<150 bp in length with an average quality score of <30) were excluded. The trimmed and unique sequences were used to define operational taxonomic units (OTUs) at the 97% similarity level. The most abundant sequence in each OTU cluster was selected as a representative sequence and was aligned using PyNAST method (Caporaso et al., 2010). Taxonomic assignment of OTUs was obtained by using the Ribosomal Database Project (RDP) pyrosequencing pipeline^2^. Taken together, the 16S rRNA gene sequencing data from 44 soil samples generated a dataset consisting of 586586 reads. After the removal of low-quality reads, 158588 reads were grouped into 24036 OTUs. The detailed 16S rRNA gene sequencing data are summarized in **Supplementary Table S2** (Supporting Information).

### Soil Physicochemical Properties Associated With Pb-Zn Tailings Contamination

As an open and heterogeneous system, soil contains heavy metals at varying concentrations. These heavy metals can be divided into ‘total’ and ‘available’ heavy metals based on geological background level or pollution source. In our study, the PbS and ZnS fractions in the Pb-Zn tailings from Sidi Village were 1.5 and 9.94%, respectively (Ning, 1992); therefore, Pb-Zn tailings have acidified soils by increasing the sulfide content of the soils (Alloway, 2013) and have continuously released heavy metals such as Pb, Zn, Cu, and Cd. The data in **Table 1** show that, in general, the levels of total and available Pb, Zn, Cu, and Cd were high in the paddy fields, followed by the citrus fields/corn fields and CKs. Moreover, the normalized values of total and available Pb, Zn, Cu, and Cd exhibited a similar trend (paddy field > citrus field/corn field > CK) (**Table 2**). These results revealed that the paddy fields were severely contaminated with heavy metals, which was also observed in our previous study (Li et al., 2015). Upon comparing citrus fields with corn fields, heavy metal concentrations were found to be associated with plant type and seemed to be higher in C4 plant-derived soil (corn field) than in C3 plant-derived soil (citrus field) due to the lower heavy metal-uptake capability of C4 plants (Greger, 2004). On the other hand, the background levels of Pb, Zn, Cu, and Cd in topsoil from Guilin, China, are 36.9, 127, 34.8, and 0.191 mg kg^−1^, respectively (Huang, 2010); however, in our study, total and available concentrations of Cd in CK soil were higher than the background values in the soil from Guilin since Pb-Zn-tailings-containing dusts settled on the surface of the CK soil during transportation, so these soils were suitable for use as control groups in our experiments.

**Table 2:** Normalized levels of heavy metals in Pb-Zn-tailings-contaminated karst soil from Sidi Village.

Type	CK	Corn field	Citrus field	Paddy field	Type	CK	Corn field	Citrus field	Paddy field
Total-Pb	0.01	0.37	0.21	0.41	DTPA-Pb	0.01	0.39	0.26	0.35
Total-Zn	0.03	0.32	0.27	0.38	DTPA-Zn	0.01	0.28	0.24	0.48
Total-Cu	0.07	0.33	0.32	0.28	DTPA-Cu	0.003	0.35	0.24	0.41
Total-Cd	0.23	0.25	0.24	0.28	DTPA-Cd	0.24	0.24	0.25	0.26
Sum	0.34	1.27	1.04	1.35	Sum	0.26	1.26	0.99	1.50

*Abbreviations are the same as those listed for **Table 1**.*

Total nitrogen, AN, pH, POC, and DOC levels were high in the paddy fields, followed by the citrus fields/corn fields and CKs. Although the highest values of SOC and CEC were observed in the paddy fields, CK also exhibited values higher than those in the corn fields and citrus fields. In karst soil, fulvic or humic acids can combine with calcium to form stable organic calcium (Cao et al., 2003). In other words, the high organic matter content of karst soil correlates with the low immobilization rate of metals (such as calcium) that can bind to, for example, fulvic or humic acids. Consequently, a high CEC in rice paddy soil implies an increased possibility of this soil binding to negatively charged anions. Of course, some elements such as Pb do not form chloride complexes due to the strong binding of these elements to organic matter (Alloway, 2013); however, these results confirm previous research that the higher the soil organic content, the higher is the metal concentration in the solid or water phase (Park et al., 2011; Deng et al., 2015).

Although MBC constitutes less than 5% of the total soil organic matter content, MBC is considered as a soil quality indicator since it plays a key role in the proper functioning of soil (Hobley et al., 2016). In our study, the MBC levels in the paddy fields, corn fields, citrus fields and CKs were 249.36, 268.37, 412.31, and 477.42 mg kg^−1^, respectively, and the MBC/SOC ratios were 0.93, 1.91, 2.13, and 2.07%, respectively. Interestingly, the higher the heavy metal content, the lower the MBC/SOC ratio was; however, no correlation was found between the heavy metal and MBC levels, as also observed by Deng et al. (2015).

**FIGURE 2:**
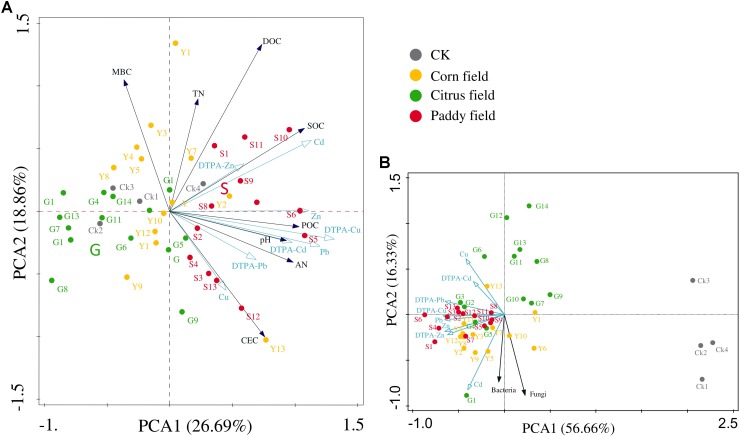
Principal component analysis (PCA) of the relationship between heavy metals (Pb, Zn, Cu, and Cd) and other soil parameters in different land-use types from Sidi Village **(A)** and the relationship between heavy metals (Pb, Zn, Cu, and Cd) and soil microbes in different land-use types from Sidi Village **(B)**. Percentage explained by the axes is shown in brackets. Abbreviations are the same as those listed for **Table 1**.

Principal component analysis was performed to better investigate the relationship between heavy metals and soil parameters (**Figure 2**). **Figures 2A,B** show that heavy metals and soil parameters were mainly concentrated in paddy fields and corn fields, indicating that rice paddy soil and corn field soil suffered from severe heavy metal contamination. Permutation tests in the PCA revealed that the distribution of heavy metals in all soil samples could be mainly expressed by the PCA1 axis (*P* = 0.01); therefore, the available Zn concentration was significantly correlated with SOC concentration (*r* = 0.307), and the POC concentration was significantly correlated with Cd (*r* = 0.354), Zn (*r* = 0.359) and available Cd (*r* = 0.317) concentrations. The results of the PCA also revealed that two groups strongly correlated with heavy metals: (i) SOC, DOC, and POC and (ii) bacterial and fungal abundance.

### Responses of Soil Microbes to Pb-Zn Tailings Contamination

Previous reports have also shown that high concentrations of heavy metals can affect the microbiota directly via changes in population size, diversity, and structure (Deng et al., 2015). In the present study, it was observed that bacterial abundances in the paddy fields were higher than those in the corn fields, CKs and citrus fields. Fungal abundances in the paddy fields were lower than those in corn fields, citrus fields and CKs. Moreover, compared with bacterial abundances, fungal abundances were mostly lower in all fields under conditions of heavy metal pollution. One explanation for these results could be that fungi are less resistant than bacteria to long-term heavy metal contamination, as reported by Jin et al. (2015) and Mossa et al. (2017). Moreover, dominant bacteria in arable fields may compete with fungi for nutrients, resulting in greater survival stress on fungal communities (Deng et al., 2015). The above results demonstrated that toxic Pb-Zn tailings generally altered soil bacterial and fungal abundance.

It should be noted that no significant correlations between bacterial abundance and heavy metal (Pb, Zn, Cu, and Cd) levels were found in our study, however, the PCA results showed the vector corresponding to bacterial abundance to be at a small angle with that corresponded to Cd levels (**Figure 2B**). Therefore, it is assumed that bacteria, which are dominant among the soil microorganisms, are less sensitive than fungi to toxic heavy metals (Clemente et al., 2006; Li et al., 2015; Zhang et al., 2016; Jia et al., 2017), as mentioned above. Therefore, in this study, we focus on the relationships between the altered bacteria and SOC in Pb-Zn-tailings-contaminated karst agricultural soils.

### Relationship Between Carbon Source Utilization and Soil Microbial Communities

**FIGURE 3:**
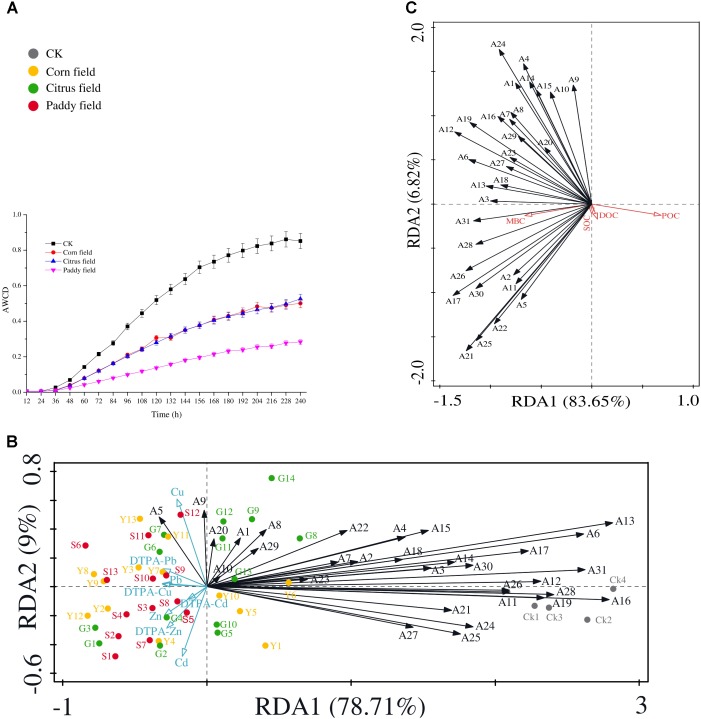
Analysis of AWCD during the 10-day incubation period (28°C) of soil samples in Biolog EcoPlates (data represent means with standard errors of three replicate plates) **(A)**; RDA biplot showing the relationship between heavy metals and 31 carbon sources **(B)**; and RDA biplot showing the relationship between 31 carbon sources and SOC, DOC, POC, and MBC **(C)**. 31 carbon sources are indicated as follows: A1, D-xylose; A2, α-D-lactose; A3, β-methyl-D-glucoside; A4, α-D-glucose-1-phosphate; A5, α-cyclodextrin; A6, glycogen; A7, D-cellobiose; A8, L-arginine; A9, L-asparagine; A10, L-phenyalanine; A11, L-serine; A12, L-threonine; A13, glycyl-L-glutamic acid; A14, pyruvic acid methyl ester; A15, Tween 40; A16, Tween 80; A17, D-galactonic acid γ-lactone; A18, *i*-erythritol; A19, D-mannitol; A20, D, L-α-glycerol phosphate; A21, phenylethylamine; A22, putrescine; A23, *N*-acetyl-D-glucosamine; A24, D-galacturonic acid; A25, D-glucosaminic acid; A26, 2-hydroxybenzoic acid; A27, 4-hydroxybenzoic acid; A28, γ-hydroxybutyric acid; A29, itaconic acid; A30, α-ketobutyric acid; and A31, D-malic acid. All data were normalized to the initial optical density at time zero; plates were read at 590 nm. Abbreviations are the same as those listed for **Table 1**.

Since the Biolog EcoPlate was introduced by Garland and Mills (1991) to assess microbial diversity and the level of sole-carbon-source utilization of environmental samples, this method has been widely used to gain insight into the relationship between soil microbial communities and carbon source switching. **Figure 3A** shows that average well color development (AWCD) in CK soil was faster than that in the other soils, and the rice paddy soils had the lowest AWCD. In addition, there was almost no difference of AWCD in soils from the corn fields and citrus fields. The variation regularity of AWCD in soils from paddy fields, corn fields and citrus fields was similar to that observed in our previous study (Li et al., 2015). This result supports our hypothesis that Pb-Zn tailings retard the rate of SOC availability in surface soils.

Moreover, RDA was performed to determine which heavy metal (Pb, Zn, Cu, or Cd) had greater effects on 31 carbon sources (**Figure 3B**) and which carbon source had more influence on the SOC pool (**Figure 3C**). The results revealed that α-cyclodextrin was closely associated with Cu and available Pb (**Figure 3B**), but no correlation was found between heavy metals and α-cyclodextrin. Cyclodextrins are cyclic oligosaccharides that can interact with appropriately sized molecules, and the hydrophobic cavities of these molecules lead to the formation of inclusion complexes; consequently metal-ion complexes with cyclodextrins can be used for molecular recognition in a wide range of applications (Li et al., 2014; Wang and Chen, 2014). Moreover, in a previous report by Zhang et al. (2015), α-cyclodextrin attached to the surface of Fe_3_O_4_@SiO_2_ nanoparticles showed adsorption capacity for Cu and Pb of more than 20 mg g^−1^, as determined by adsorption experiments. It is reasonable to assume that the remained α-cyclodextrin in soil systems can decrease SOC availability. L-asparagine is also closely associated with Cu and POC concentrations, as shown in **Figures 3B,C**. The results could possibly be explained by the previous study that copper ions can promote the crystal growth of L-asparagine (Santana et al., 2016). Therefore, due to the presence of toxic heavy metals and retarded AWCD, the POC/SOC ratio, which represents the contribution of POC to SOC (Nie et al., 2016), was lower in the sampled soils (POC/SOC ratio was 0.018, 0.025, 0.034, and 0.027% in the CK, corn field, citrus field and paddy field samples, respectively). In addition, malic acid which is a highly biodegradable low-molecular-weight organic compound (Chowdhury et al., 2014) in soil exhibited significant negative correlation with those of Pb (*r* = −0.486), Zn (*r* = −0.433), available Pb (*r* = −0.547), and available Cu (*r* = −0.495), which can be explained by the RDA biplot in **Figure 3B**. Moreover, the vector corresponding to malic acid, a commonly observed organic acid, showed a small angle compared with that corresponding to MBC concentration (significant correlation, *r* = 0.316) (**Figure 3C**). Based on the significantly positive correlations between α-cyclodextrin and L-asparagine (*r* = 0.597), α-cyclodextrin and malic acid (*r* = 0.454), and L-asparagine and malic acid (*r* = 0.519), we hypothesize that the α-cyclodextrin, L-asparagine and malic acid utilization rates up- or down regulate the variance in SOC availability.

In addition, to better investigate the ecological characteristics of carbon source utilization, five diversity indices of microbial communities were calculated from the Biolog Eco data (**Table 3**). The highest McIntosh index, Shannon-Wiener diversity index, Simpson’s diversity index and species richness were found for the CKs, followed by citrus fields, corn fields, and paddy fields, while the lowest Shannon’s evenness index was found for the CKs, followed by citrus fields, corn fields, and paddy fields. In our previous study (Li et al., 2015), we reported that the ecological diversity of soil microbial communities varied similarly in citrus fields, corn fields and paddy fields. In this respect, the ecological diversity of soil microbial communities reflected a decrease in soil microbial activity, which could be ascribed to high doses of heavy metals. Moreover, the decreased McIntosh index, Shannon-Wiener diversity index, Simpson’s diversity index and species richness of the CKs, citrus fields, corn fields and paddy fields revealed that microbial community structure tended to be simple; therefore, the highest Shannon’s evenness index was obtained for paddy fields. It should be noted that the Shannon’s evenness index, McIntosh index, Shannon-Wiener diversity index and Simpson’s diversity index of the CKs and paddy fields were clearly different, but the species richness was not. This finding might indicate that alterations in microbial communities affect the variance in SOC availability.

**Table 3:** Ecological diversity of microbial communities based on degree of carbon source utilization.

	*U*	*H*	*D*	*E*	*S*
CK	5.68 ± 0.26 A^∗^	3.16 ± 0.03 A	0.95 ± 0.00 A	0.98 ± 0.01 A	25.50 ± 1.04 A
Corn field	3.65 ± 0.45 AB	2.90 ± 0.08 AB	0.93 ± 0.01 AB	1.06 ± 0.04 A	18.23 ± 2.01 BC
Citrus field	4.19 ± 0.74 AB	2.93 ± 0.08 A	0.93 ± 0.01 AB	1.05 ± 0.03 A	19.00 ± 1.99 B
Paddy field	2.51 ± 0.21 B	2.65 ± 0.06 B	0.91 ± 0.01 B	1.11 ± 0.04 A	12.15 ± 1.30 C

*^∗^The data are means ± standard deviations (13 paddy soil samples, 13 corn field soil samples, 14 citrus soil samples, and 4 CK soil samples). Statistical pairwise multiple comparisons of data homogeneity were carried out by the Tukey test: means with the same letter in the same column are not significantly different at P < 0.05. U, McIntosh index; H, Shannon-Wiener diversity index; D, Simpson’s diversity index; E, Shannon’s evenness index; and S, species richness.*

### Relationship Between Microbial Taxa and SOC Availability

**FIGURE 4:**
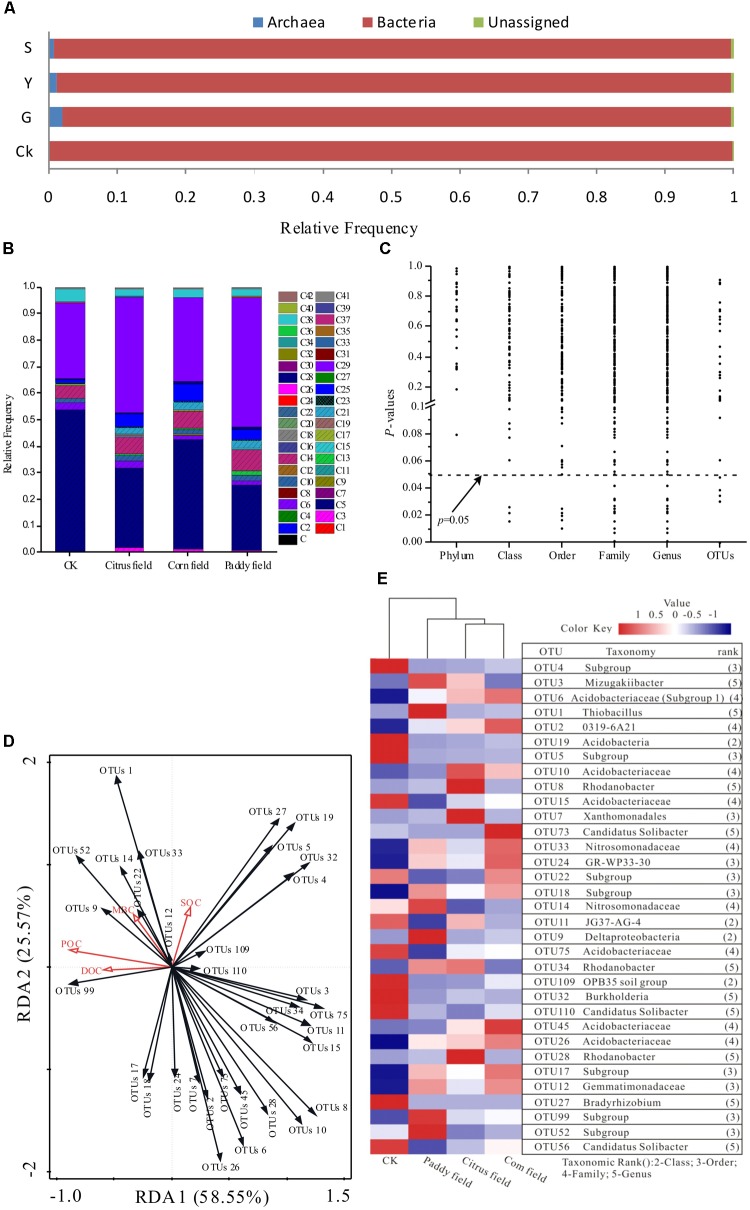
Effects of Pb-Zn tailings on the taxonomic and functional profiling of microbial communities identified via high-throughput 16S rRNA gene sequencing from karst soil in Sidi Village. **(A)** Relative abundances of bacterial, archaeal and unassigned communities; **(B)** relative frequencies of dominant phyla (16S rRNA gene datasets); **(C)** *P*-values of the relationship between SOC levels and relative taxa frequencies at different taxonomic levels; **(D)** RDA plot used to show the relationship between SOC, DOC, POC, MBC and the 33 most abundant OTUs; and **(E)** heat map illustrating the relative frequencies of the 33 most abundant OTUs based on the V3–V5 region of the 16S rRNA gene sequences. The dominant phyla are indicated as follows: C, Euryarchaeota; C1, MCG; C2, MEG; C3, Thaumarchaeota; C4, DHVEG-6; C5, Acidobacteria; C6, Actinobacteria; C7, Aerophobetes; C8, Aminicenantes; C9, Armatimonadetes; C10, Bacteroidetes; C11, Candidate division OP3; C12, Chlamydiae; C13, Chlorobi; C14, Chloroflexi; C15, Cyanobacteria; C16, Deinococcus-Thermus; C17, Elusimicrobia; C18, Fibrobacteres; C19, Firmicutes; C20, GOUTA4; C21, Gemmatimonadetes; C22, Hydrogenedentes; C23, Latescibacteria; C24, Microgenomates; C25, Nitrospirae; C26, Omnitrophica; C27, Parcubacteria; C28, Planctomycetes; C29, Proteobacteria; C30, SHA-109; C31, SM2F11; C32, Saccharibacteria; C33, Spirochaetae; C34, TA06; C35, TM6; C36, Tenericutes; C37, Thermotogae; C38, Verrucomicrobia; C39, WCHB1-60; C40, WD272; C41, other; and C42, unassigned.

Up to 10,000 distinct species of soil prokaryotes have been found to coexist in a single soil sample (Miyashita, 2015); however, only a few species occur at high frequencies (Sogin et al., 2006). Thus, most (over 99%) of the sequences obtained based on the V3–V5 region of 16S rRNA tag of prokaryotic communities were assigned to 41 phyla (**Supplementary Table S2** and **Figure 4B**). Acidobacteria and Proteobacteria dominated the prokaryotic communities studied by high-throughput 16S rRNA gene sequencing. Acidobacteria accounted for 53.78, 29.69, 41.10, and 24.67% of the total readings (mean relative frequency) in CKs, citrus fields, corn fields and paddy fields, respectively, and Proteobacteria accounted for 28.40, 43.41, 31.23, and 48.79%, respectively. In addition, Verrucomicrobia, Chloroflexi, Actinobacteria, Bacteroidetes, and Nitrospirae were also major phyla present in these soils. Proteobacteria and Acidobacteria have also been found to be the most prominent soil phyla in the world, accounting for 70 ∼ 80% of all soil bacteria (García-Fraile et al., 2016). In addition, Miyashita (2015) found that Acidobacteria (32.3% of the total soil bacterial population, including unclassified bacteria) and Proteobacteria (29.0% of the total soil bacterial population) were the dominant soil phyla in tropical areas of Southeast Asia. Interestingly, Acidobacteria were accounting for 50% of the total soil bacterial population in acidic soils (García-Fraile et al., 2016); however, they accounted for 53.78, 29.69, 41.10, and 24.67% in the CKs, citrus fields, corn fields and paddy fields with pH ≤ 5.91 in our study, as revealed via high-throughput 16S rRNA gene sequencing. In summary, Acidobacteria and Proteobacteria are the most prevalent phyla in CKs, citrus fields, corn fields and paddy fields and play a fundamental role in soil environments.

Mine tailings usually lead to extreme ecological conditions (Wong, 2003). With the exception of certain bacteria that tolerate these extreme conditions, most extremophiles belong to the kingdom archaea (Konings et al., 2002). It was found that the V3–V5 region of the 16S rRNA gene could provide unprecedented insight into bacterial and archaeal communities from wetland soils (Narrowe et al., 2017). In this context, bacterial, archaeal and unassigned communities were found in CKs, citrus fields, corn fields and paddy fields (**Figure 4A**). The archaeal communities in CKs, citrus fields, corn fields and paddy fields constituted 0.08, 1.89, 1.21, and 0.78%, respectively, of the total reads (mean relative frequency). This result was supported by the fact that most of the archaeal taxa showed very low relative frequencies in soils with less heavy metal contamination (CK), which has also been shown for microbial communities in acid mine drainage (Chen et al., 2015). Moreover, Euryarchaeota, a phylum within the archaea, had mean relative frequencies of 0.15, 0.08, and 0.13% in citrus fields, corn fields and paddy fields, respectively. As a result, almost no Euryarchaeota communities were present in CKs, possibly due to their different niche selection.

The effects of toxic Pb-Zn tailings on SOC availability were primarily studied based on structural changes in the prokaryotic communities as well as POC, DOC, and MBC variance. It was found that the variance in POC, DOC, and MBC availability was independent of microbial community, as revealed via high-throughput 16S rRNA gene sequencing. To better study these effects, *P*-values for taxonomic relative abundance patterns of microbial communities based on 16S rRNA gene sequence analysis as well as SOC concentrations in the surface soils were determined (**Table 1** and **Figure 4**). Although the relative frequencies of bacterial and archaeal phyla changed under the effect of Pb-Zn tailings in the soil samples (**Figure 4A**), the values remained unchanged under conditions of variance in SOC availability (**Figure 4C**). In other words, the soil microbes identified via high-throughput 16S rRNA gene sequencing at the phylum level were unaffected by SOC mineralization. However, the patterns of their class, order, family, genus or OTUs changed with variation in SOC availability (**Figure 4C**). We assumed that the effects of Pb-Zn tailings on variance in SOC availability may be the consequence of a series of changes in microbial community functions (>0.5%) at the class, order, family, genus and OTU levels revealed via high-throughput 16S rRNA gene sequencing. Although only 1% of the OTUs (the lowest taxonomic level, 16S rRNA gene) were associated with variance in SOC availability due to toxic Pb-Zn tailings (*P* ≤ 0.05, **Figure 4B**), the contribution of these OTUs to variance in SOC availability should not be ignored. To better understand the role of these bacteria with agricultural soil types, a heat map of the 33 most abundant OTUs was constructed for all soil samples (**Figure 4E**). The heat map clearly showed that Acidobacteria- and Proteobacteria-related OTUs were widely distributed in all soil samples. Bacteria-related OTUs 4, 5, 15, 19, 27, 32, 56, 75, 109, and 110 were at the high relative abundance in CKs. In contrast, several OTUs were at the high relative abundance in Pb-Zn-tailings-contaminated soils. For example, the bacteria-related OTUs 1, 3, 9, 14, 52, and 99 were at the high relative abundance in paddy fields and the bacteria-related OTUs 12, 17, 18, 24, and 33 were at the high relative abundance in paddy fields and corn fields. Among these most frequent OTUs that could be classified at the genus level, OTU 1 (Thiobacillus), OTU 3 (Mizugakiibacter), OTU 27 (Bradyrhizobium) and OTU 32 (Burkholderia) were classified as Proteobacteria, while two OTUs (56 and 110) related to Candidatus Solibacter were classified as Acidobacteria; these OTUs might play an important role in SOC availability.

Thiobacillus, which are sulfur-oxidizing bacteria (Verástegui-Valdés et al., 2014), are abundant in post-mining soils (de Quadros et al., 2016) and are known to oxidize a range of reduced sulfur compounds (H_2_S, FeS_2_, S_2_O_3_^2-^ and polythionate) using O_2_ or NO_3_^−^ as an electron acceptor (Wörner et al., 2016). Based on the geological survey of Pb-Zn deposits in Sidi Village, PbS (1.5%) and ZnS (9.94%) are the principal components of these deposits (Ning, 1992). In this respect, the relatively high frequency of the sulfur-oxidizing bacteria Thiobacillus affiliated with paddy fields is probably associated with the large amount of Pb-Zn tailings (sulfur compounds) in the soil (Verástegui-Valdés et al., 2014), which is supported by the total analytical results for Pb, Zn, Cu, and Cd in this study (**Table 1**). Moreover, it was reported that aerobic Thiobacillus bacteria could act as denitrifiers under anoxic conditions, especially in rice paddy soils (Embarcadero-Jiménez et al., 2016). Therefore, Thiobacillus, being a part of the oxidative branch of sulfur cycling in rice paddy soils, produce compounds that are toxic to other microbial communities (Wörner et al., 2016) and serve as chemoautotrophic contributors to the SOC pool. According to the Pearson correlation between the Thiobacillus OTUs and the levels of heavy metals, Thiobacillus OTUs correlated significantly with total Pb (*r* = 0.260), total Zn (*r* = 0.519), total Cd (*r* = 0.435), available Zn (*r* = 0.631), available Cu (*r* = 0.373) and available Cd (*r* = 0.534) levels. Therefore, due to the presence of toxic heavy metals, Thiobacillus might have retarded the ability of other microbes to degrade SOC and contribute organic debris to the SOC pool, which in turn led to high SOC concentrations in the rice paddy soil. In this respect, the results of the RDA also showed significant correlation between Thiobacillus abundance and SOC concentration (*r* = 0.296) as well as POC concentration (*r* = 0.431) (**Figure 4D**). Moreover, Cao et al. (2017) reported positive associations between Thiobacillus and carbon fractions in sediment soil from China.

The growth of Mizugakiibacter, which are heterotrophic bacteria belonging to the family *Xanthomonadaceae* in the class Gammaproteobacteria, was observed in the temperature range 25 ∼ 52°C and at pH 5.0 ∼ 8.2 (optimal growth was observed at 48 ∼ 50°C and at pH 6.0 ∼ 7.0) by Kojima et al. (2014). During our sampling time, the near-surface air temperature was 36°C, and the soil pH varied between 5.18 and 5.91. In addition, Mizugakiibacter were at the high relative abundance in rice paddy soil and citrus field soil. Based on the results, it is reasonable to assume that Mizugakiibacter are facultatively anaerobic. In a previous report, switching the growth of Mizugakiibacter from an aerobic to an anoxic environment increased nitrate consumption and nitrite production (Kojima et al., 2014), which might contribute to the TN levels. The Pearson correlation illustration showed weak correlations between Mizugakiibacter abundance and TN concentration (*r* = −0.037) as well as SOC concentration (*r* = 0.040). In addition, Mizugakiibacter are sensitive to heavy metals (Guo et al., 2017). In our study, we expand on our previous work (Jin et al., 2015; Li et al., 2015) to reveal a strong association between Mizugakiibacter and heavy metals. The correlations between Mizugakiibacter abundance and the levels of Zn, Cu, Cd, available Pb, available Zn, available Cu and available Cd (*r* = 0.027, 0.112, −0.138, 0.247, −0.020, 0.203, and −0.104, respectively) were not significant; however, Mizugakiibacter abundance was significantly correlated with Pb levels (*r* = 0.312). Indeed, the results are similar to previous findings (Guo et al., 2017) that Mizugakiibacter abundance was negatively correlated with the levels of exchangeable Pb (*P* < 0.01). Therefore, coupled with the influence of Thiobacillus, the availability of soil organic matter decreased and the SOC was retained in the rice paddy soil.

Bradyrhizobium, is involved in ammonia production and is best known for its role as a nitrogen-fixing symbiont on legume roots (Zhalnina et al., 2013), playing an important role in agricultural productivity and global nitrogen cycling (VanInsberghe et al., 2015). In our study, Bradyrhizobium is negatively correlated with AN and TN levels (*r* = −0.164 and −0.135) and at the high relative abundance in CKs (non-agricultural soils), as previously reported by Zhalnina et al. (2013) for soil samples from agricultural and non-agricultural area in United Kingdom and United States. This result led to one question. Why is the Bradyrhizobium so low in the agricultural soils (citrus field, corn field and paddy field) compared to non-agricultural soils (CKs)? It was reasonable to assume that higher levels of N would reduce the role for nitrogen-fixing organisms in non-legume agriculture (Zhalnina et al., 2013). As a result, the number of free-living bradyrhizobia declines since fixed N inhibits N_2_ fixation in citrus field, corn field and paddy field. In addition, the cycling rate of a large percentage of the Earth’s active carbon strongly was associated with the availability of nitrogen; consequently the Bradyrhizobium not only affected nitrogen fixation, but also influenced the availability of labile organic carbon fractions (weak negative correlations between Bradyrhizobium and DOC concentration [*r* = −0.190] as well as POC concentration [*r* = −0.173]) and the SOC retention (*r* = 0.163), which was also exhibited by the RDA biplot in **Figure 4D**. Though no previous work shows the dramatic effects of heavy metals on Bradyrhizobium, it was found that Bradyrhizobium abundance was negatively correlated with the levels of Pb, Zn, Cu, Cd, available Pb, available Cu and available Cd (*r* = −0.421, −0.559, −0.284, −0.342, −0.559, −0.542, and −0.464, respectively). The observation that these effects are obvious, as seen through **Figures 4D,E**, is also novel. All of these results taken together with the results presented here are expected to encourage an examination of free-living Bradyrhizobium in unpolluted soils to determine their ability on SOC-nitrogen availability.

Burkholderia are well known for their ability to consume organic matter from root exudates (Weber and King, 2012). For example, Burkholderia containing ligninolytic genes are capable of degrading lignin (Bugg et al., 2011a,b). Moreover, **Figure 4D** shows that Burkholderia abundances had long vector angles and were located quite far away from the POC and DOC vectors; however, Burkholderia abundance exhibited a weak negative correlation with POC concentration (*r* = −0.191) and DOC concentration (*r* = −0.196). Therefore, the low average relative abundance of Burkholderia in the citrus, corn and paddy fields might have decreased SOC availability, which can be explained by the RDA biplot with short vector angles and close associations between Burkholderia abundance and SOC levels, although no significant correlation existed between the two (*r* = 0.145). In addition, Burkholderia abundance was significantly negative correlated with the levels of Pb (*r* = −0.382), Zn (*r* = −0.478), available Pb (*r* = −0.477), available Cu (*r* = −0.468), and available Cd (*r* = −0.472). The results presented here demonstrate that Burkholderia are at the high relative abundance in CKs. In this respect, based on the small vector angle seen in **Figure 4D**, Burkholderia abundance was significantly positively correlated with that of Bradyrhizobium (*r* = 0.814).

Candidatus Solibacter can produce enzymes that breakdown organic carbon (Pearce et al., 2012) and are also known to be adapted to low-nutrient conditions (Eichorst et al., 2011). In CKs, the average AN concentration was 92.47 mg kg^−1^, which was lower than the AN concentration of citrus fields, corn fields and paddy fields. The low-nutrient adaptations of the bacteria-related OTUs 56 and 110 were congruent with the greater relative frequency of these OTUs in CK soils. Since OTUs 56 and 110 were classified as Acidobacteria, the ability of these OTUs to degrade organic carbon compounds might have allowed Acidobacteria to displace other bacterial species that were unable to survive in the presence of these substrates, particularly at low nutrient levels (García-Fraile et al., 2016). Consequently, large vector angles between the abundance of OTU 56, the abundance of OTU 110, as well as DOC, POC, MBC, and SOC levels are shown in the RDA biplot in **Figure 4D**; however, there was weak correlation between these parameters (*P* > 0.05) with the exception of the significantly negative correlation between the abundance of OTU 56 and DOC concentration (*r* = −0.311). Previous studies have revealed that Candidatus Solibacter can provide environmental conditions that are beneficial to other bacteria involved in the degradation of complex organic compounds (Rime et al., 2015), which are able to degrade more recalcitrant organic materials (Mancera-Lopez et al., 2007); consequently, the low abundance of Candidatus Solibacter in rice paddy soil severely contaminated with Pb-Zn tailings decreased SOC availability, suggesting that the highest SOC concentration was in rice paddy soils.

Among the other most frequent bacteria-related OTUs that were not classified at the genus level, six OTUs (4, 5, 15, 19, 75, and 109) were at the high relative abundance in the CK environment, four OTUs (9, 14, 52, and 99) were at the high relative abundance in rice paddy soils, and five OTUs (12, 17, 18, 24, and 33) were at the high relative abundance in paddy soils and corn fields. In addition, these OTUs were classified as Acidobacteria (OTUs 4, 5, 15, 17, 18, 19, 52, 75, and 99), Proteobacteria (OTUs 9, 14, 24, and 33), Gemmatimonadetes (OTU 12) and Verrucomicrobia (OTU 109). In addition, Gemmatimonadetes and Verrucomicrobia also play important roles in soil systems (Gittel et al., 2014). For example, Gemmatimonadetes (OTU 12) can enhance the cycling of essential micro- or macronutrients, which may partially improve soil fertility (Lesaulnier et al., 2008) and is reflected by the high abundance of Gemmatimonadetes in rice paddy soils and corn soils, as seen in **Figure 4E**. On the other hand, Verrucomicrobia (OTU 109), as less well-known soil microorganisms specializing in methanol oxidation, represent an important but under studied mechanism by which organic carbon is transferred from surface litter layers to mineral soils (Fierer, 2015).

**FIGURE 5:**
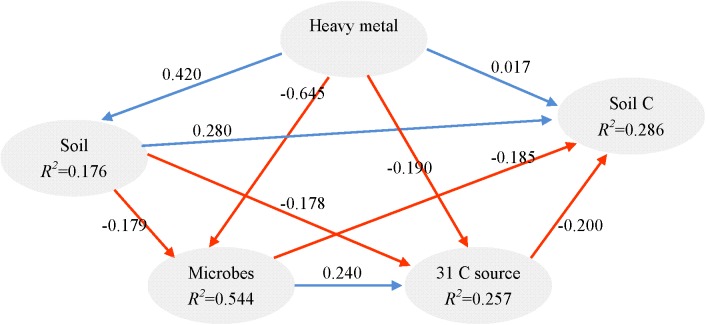
Directed graph of the PLS-PM of heavy metal (total and DTPA-extractable Pb, Zn, Cu, and Cd) effects on soil (pH, AN, CEC, and TN), soil C (SOC, TOC, POC, and MBC), 31 carbon source and microbial communities (the 33 most abundant OTUs). The path coefficients and the explained variability (*R*^2^) were calculated after 999 bootstraps. Blue solid arrows indicate positive direct effects, and red solid arrows indicate negative direct effects. Models with different structures were assessed using the Goodness of Fit (GoF) statistic, a measure of the overall prediction performance. For the model represented here, the GoF was 0.363.

It is known that space limitations determine specific relationships (for example interference, competition and convergence) between coexisting microbes in soils (Roesch et al., 2007). We found that the abundance of OTU 1 (Thiobacillus) was significantly negatively correlated with that of OTUs 15, 56 (Candidatus Solibacter) and 75 (*r* = −0.310, −0.285, and −0.354); the abundance of OTU 3 was significantly negatively correlated with that of OTU 14 (*r* = −0.338); and the abundance of OTU 99 was significantly negatively correlated with that of OTUs 19 and 32 (*r* = −0.264 and −0.295). The results suggest that binding competition or interference might be involved. In contrast, there were significant positive correlations among the abundances of other OTUs (1, 4, 5, 9, 12, 14, 15, 17, 18, 19, 24, 27, 32, 33, 75, 99, 109, and 110) (*P* < 0.05), as seen in **Supplementary Table S3** (**Supplementary Data**), which indicate that these OTUs can probably coexist in soil due to similarity, convergence and divergence adaptations. These relationships are also evidenced by the RDA biplot and heat map shown in **Figures 4D,E**, respectively. Moreover, we also discovered that L-asparagine utilization profiles showed significant positive correlation with the abundances of OTUs 24, 75, and 109 (*r* = 0.383, 0.350, and 0.292, respectively), and malic acid utilization profiles showed significant positive correlation with the abundances of OTUs 4, 5, 19, 27 (Bradyrhizobium), 32 (Burkholderia), 75 and 109 (*r* = 0.286, 0.361, 0.387, 0.384, 0.363, 0.285, and 0.301, respectively). Here, we can infer that these bacteria controlled the carbon source utilization rate and variance in SOC availability, as revealed by the carbon source utilization chart, *P*-values and heat map shown in **Figures 3**, **4C**, **5**, respectively. Interestingly, uncultured Deltaproteobacteria GR-WP33-30 (OTU24), which can live in aerobic/anoxic Pb-Zn-tailings-contaminated soils, presented the key to understanding the intrinsic relationships between soil microorganisms and SOC cycles. These findings are consistent with other studies that GR-WP33-30 was identified as keystone genera in karst rocky desertification area (Ma et al., 2016; Xue et al., 2017). Consequently, future work is needed to better understand the role of the bacterium in karst SOC mineralization. In other words, SOC mineralization relies on these microorganisms after long-term acclimation in heavy metal-contaminated soils. These findings explain why Kemmitt et al. (2008) and Brookes et al. (2017) did not observe a dependence of SOC mineralization on the size, community structure or specific activity of soil microorganisms in simulation experiments, a result with which Kuzyakov et al. (2009) have disagreed.

### PLS-PM and PCoA Performed on 16S rRNA Gene Sequencing Data

It is known that redundant heavy metals often have deleterious effects on microbes (Xu et al., 2012). In order to explore the key drivers shaping soil microbial communities and test our hypotheses, we supplied comprehensive proof by using a variety of statistical methods. The partial Mantel test showed significant effects of heavy metals on the microbial community structure (*p* < 0.01), no matter which edaphic factors were controlled (**Table 4**). Moreover, the PLS path model was constructed to integrate the complex interrelationships among edaphic factors and microbial communities (**Figure 5**). According to the PLS-PM, heavy metals exerted significantly negative direct effects on microbial communities (*r* = 0.296, *p* < 0.01) and significantly positive direct effects soil properties (*r* = 0.171, *p* = 0.010). Our findings suggest that Pb-Zn-tailings-contamination determined soil microbial composition. There was no significant direct effect of heavy metals on soil C and 31 carbon source (*p* > 0.05). Instead, there was a negative direct effect of microbial communities on soil C, but not significant (*p* > 0.05). Interestingly, OTU 24 exerted significant direct effects on 31 carbon source (*r* = 0.278, *p* = 0.007), but not the soil C (*p* > 0.05). Together, this suggested that heavy metals exerted negative effects on soil C cycle mainly through altered microbial communities, similar to the previous report that cations decrease microbial access to SOC and mineralization (Bronick and Lal, 2005).

**Table 4:** The influence of heavy metals on microbes by partial Mante test.

Effect of	Heavy metal^*a*^							

Controlling for		Soil^*b*^	Soil C^*c*^	31 carbon source	Soil C and 31 carbon source	Soil and soil C	Soil and 31carbon source	Soil, soil C and 31 carbon source
	*r*	*r*	*r*	*r*	*r*	*r*	*r*	*r*
Microbes	0.295	0.310	0.296	0.295	0.296	0.310	0.310	0.310

*All p-values are less than 0.01. Heavy metal^a^, total and DTPA-extractable Pb, Zn, Cu, and Cd; Soil^b^, pH, AN, CEC and TN; Soil C^c^, SOC, TOC, POC, and MBC.*

**FIGURE 6:**
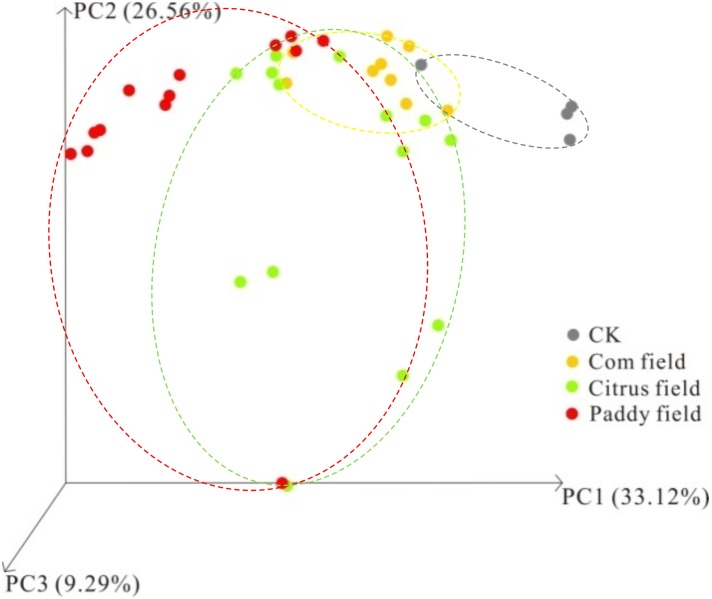
Bray–Curtis dissimilarity PCoA based on 16S rRNA gene sequencing data.

To further analyze how the distribution patterns of microbial communities altered by toxic Pb-Zn tailings, Bray–Curtis-dissimilarity-based PCoA was performed on 16S rRNA gene sequencing data. PCoA demonstrated that PC1, PC2, and PC3 accounted for 68.97% of the observed variance (**Figure 6**). The analysis was limited to the components PC1, PC2, and PC3 because these components accounted for the separation of the classes, as explained later. In this respect, the PCoA showed that no overlap of the microbial taxa was revealed via high-throughput 16S rRNA gene sequencing from rice paddy soil and CKs, although an overlap existed in the soil samples from citrus fields, corn fields and paddy fields as well as in the soil samples from citrus fields, corn fields and CKs, indicating that the effect of toxic Pb-Zn tailings on the soil microbial distribution pattern revealed via high-throughput 16S rRNA gene sequencing depended on the heavy metal concentration, which have been explained by **Figure 5**. Therefore, the above results suggest a significant difference in the distribution patterns of microbial communities in paddy fields and CKs, which is consistent with the observed relationship between heavy metals and soil parameters as well as ecological diversity and taxonomic and functional profiling of microbes in paddy fields and CKs, as seen in **Table 3** and **Figures 3**, **6**. These interpretations of the PCoA results for paddy fields and CKs also suggest that the altered levels of microbes depend on the heavy metal concentrations. Taken together, all the experimental results presented in this study clearly support our assertions that the altered structure and/or function of microbes, especially some facultative soil bacteria, after long-term acclimation in heavy-metal-contaminated soil serve as the key to exploring the effect of long-term heavy metal contamination (almost 40 years) on SOC availability in surface karst soil.

## Conclusion

Our results reveal that the paddy fields suffered from severe Pb-Zn-tailings contamination, followed by the corn/citrus fields and control groups, and the SOC concentration in the paddy fields was the highest. The microbial diversity and sole-carbon-source utilization profiles indicated that, compared to corn fields, citrus fields and CKs, the variance in SOC availability, McIntosh index, Shannon-Wiener diversity index, Simpson’s diversity index and species richness of rice paddy soils were the lowest. Moreover, all soil samples were dominated by Acidobacteria and Proteobacteria. Among the most frequent bacteria-related OTUs, L-asparagine utilization profiles showed significant positive correlation with the abundances of OTUs 24, 75, and 109, and malic acid utilization profiles showed significant positive correlation with the abundances of OTUs 4, 5, 19, 27, 32, 75, and 109, indicating that these bacteria might play important roles in mediating the SOC mineralization process. In addition, due to the important role of uncultured Deltaproteobacteria GR-WP33-30 (OTU 24) that can live in aerobic/anoxic soils, the SOC concentration in the rice paddy fields was the highest.

Moreover, probably the most significant conclusion of this study is that the class, order, family, genus and OTUs of soil microbes revealed via high-throughput 16S rRNA gene sequencing changed with the variation in SOC availability within their taxonomic levels, which is in contrast to the regulatory gate hypothesis. Surprisingly, some bacteria, after long-term acclimation in heavy metal-contaminated soils, control the labile carbon source utilization rate and SOC availability in surface karst soils; however, the mechanism of these bacteria regulating labile organic carbon components remains unclear. In summary, this work expands our understanding of the effect of these microbes on SOC availability in karst soil systems and emphasizes their function on SOC sequestration. The results might have important implications for future investigations related to global soil-carbon sequestration and storage via changes in microbial community structure (see, e.g., Liang et al., 2017). Accordingly, to fully appreciate the effect of these soil microbes on SOC components (for example, labile organic carbon components), further experimental research (DNA-SIP) should be conducted to study the rate-limiting reaction step of SOC availability and the turnover of organic carbon and nitrogen in soil. In addition, the relationships between other soil microbes (for example, archaea) and toxic Pb-Zn tailings should also be considered.

## Author Contributions

QL, XW, ZJ, JC, and WM designed the study. QL, CL, ZJ, and AS collected the soil samples. QL, CL, AS, and YL conducted the experiments and data analysis. QL, XW, and WM wrote the paper. All authors critically commented on and contributed to the manuscript.

## Conflict of Interest Statement

The authors declare that the research was conducted in the absence of any commercial or financial relationships that could be construed as a potential conflict of interest.

## Footnotes

1 http://www.gastonsanchez.com/PLS

2 http://pyro.cme.msu.edu/

## Supplementary Material

TABLE S1 The details soil parameters of Pb-Zn-tailings-contaminated karst soil in Sidi Village. **(A)** AN, CEC, TN, SOC, DOC, POC, MBC and pH of Pb-Zn-tailings-contaminated karst soil in Sidi Village; **(B)** The concentration of Pb, Zn, Cu, and Cd in Sidi Village; **(C)** Soil bacterial and fungal abundance in Sidi Village; **(D)** The utilization profiles of 31 carbon sources in Sidi Village.

TABLE S2 Sequencing information at the phylum, class, order, family, genus and OTU levels.

TABLE S3 Correlative analysis of the 33 most abundant OTUs and 31 carbon sources.
